# *Notes from the Field*: Dengue Outbreak — Naoero, 2025

**DOI:** 10.15585/mmwr.mm7532a2

**Published:** 2026-08-20

**Authors:** Liliana Sánchez-González, Dezheen Zebari, Marquita Detenamo, Susana Nakalevu, Christal Teabuge, Iivan Aingimea, Viachaslau Markau, Andrew Samuel, Gilberto A. Santiago, Laura E. Adams, Gabriela Paz-Bailey, Angelique Makutu

**Affiliations:** ^1^Dengue Branch, CDC, San Juan, Puerto Rico; ^2^Republic of Naoero Hospital, Denigomodu, Naoero; ^3^Department of Public Health, Denigomodu, Naoero.

**Summary**

**What is already known about this topic?**

Eleven Pacific Island countries, including Naoero (population approximately 11,000), reported dengue outbreaks in 2025. Before 2025, no dengue cases had been confirmed in Naoero since 2018.

**What is added by this report?**

In 2025, a total of 1,402 suspected dengue cases (477 of which were confirmed), were reported in Naoero; young children were disproportionately affected, including two pediatric deaths. The outbreak response required a rapid and substantial redirection of public health and clinical resources and supplies.

**What are the implications for public health practice?**

Strengthening dengue prevention through the expansion of vector control activities and community engagement, improving early detection through surveillance and laboratory testing, and ensuring readiness for pediatric dengue clinical management are critical to reducing morbidity and health system strain during dengue outbreaks.

## Introduction

Dengue is a febrile disease caused by one of four dengue viruses (DENV-1–4); it is primarily transmitted through the bite of infected *Aedes* species mosquitoes. In 2025, 11 of 21 Pacific Island countries and areas reported dengue outbreaks (*1*). Naoero, a Pacific Island country with a population of approximately 11,000, reported dengue outbreaks in 2014 (DENV type unknown; 251 cases and no deaths), 2017 (DENV-2; 964 cases and three deaths), and 2018 (DENV-1; 114 cases and no deaths) (*2*). No cases were reported during 2019–2024.

On June 27, 2025, a boy aged 16 years was evaluated for fever, headache, myalgia, and arthralgia at the Republic of Naoero Hospital (RoNH), the island’s only hospital, which has six emergency department beds and 65 inpatient beds. Dengue was suspected based on signs and symptoms and ongoing regional outbreaks. The patient received a positive dengue nonstructural protein 1 (NS1) antigen result from a rapid diagnostic test (RDT) that assesses dengue NS1, immunoglobulin M (IgM), and immunoglobulin G (IgG). The next day, a boy aged 17 years was evaluated for fever, headache, and vomiting and received a positive dengue IgM RDT result.

## Investigation and Outcomes

### Outbreak Investigation

**Case definitions.** After identification of the two initial cases, clinicians were alerted and advised to suspect dengue and to administer the available dengue RDT to patients with dengue signs or symptoms (*3*). Patients with signs and symptoms of dengue, (fever, nausea, vomiting, rash, aches and pains, and leukopenia), dengue warning signs (abdominal pain or tenderness, persistent vomiting, fluid accumulation, mucosal bleeding, lethargy or restlessness, hepatomegaly, or increasing hematocrit), or signs of severe dengue (severe plasma leakage, severe bleeding, or severe organ involvement), in conjunction with a positive NS1 or IgM (or both) RDT result, were considered to have laboratory-confirmed dengue. Patients with dengue symptoms and who received negative NS1 and IgM RDT results were considered to have suspected dengue. For clinical management purposes, patients with suspected dengue were treated as having dengue, given the potential for false negative RDT results. Within 10 days, 32 new laboratory confirmed cases were identified.

**Declaration of dengue outbreak and outbreak findings.** The government of Naoero declared a dengue outbreak on July 8, 2025. During June 27–September 28, a total of 1,402 persons with suspected dengue were identified and received testing; 477 (34%) cases were laboratory-confirmed (Figure) (Supplementary Table). Among confirmed cases, 252 (53%) occurred in males; the median patient age was 10 years (range = 0–70 years). Most confirmed cases (375; 79%) occurred in persons aged ≤19 years, with 157 (33%) in children aged 5–9 years. Overall, 114 (24%) patients required hospitalization; among the 477 patients with confirmed dengue, two pediatric deaths (0.42%) were reported. Confirmed cases were reported from all 14 Naoero districts. On August 16, a total of 81 samples were sent for testing at CDC by RoNH. Among those, DENV-2 was identified in 22 (58%) of the 38 RDT-positive samples and in four (9%) of the 43 RDT-negative samples tested; sample degradation might have precluded identification of DENV in the remaining positive samples and prevented direct evaluation of the RDT performance in this setting. Phylogenetic analysis of sequences from specimens obtained from 12 patients confirmed circulation of the DENV-2 cosmopolitan genotype, lineage 5 (clade 2II_F) (*4*). The number of confirmed dengue cases began to decline during the week of August 22, and the last confirmed case was identified on September 18. The government of Naoero declared the outbreak over on September 28, 2025. This activity was reviewed by CDC, deemed not research, and conducted consistent with applicable federal law and CDC policy.*

**FIGURE:**
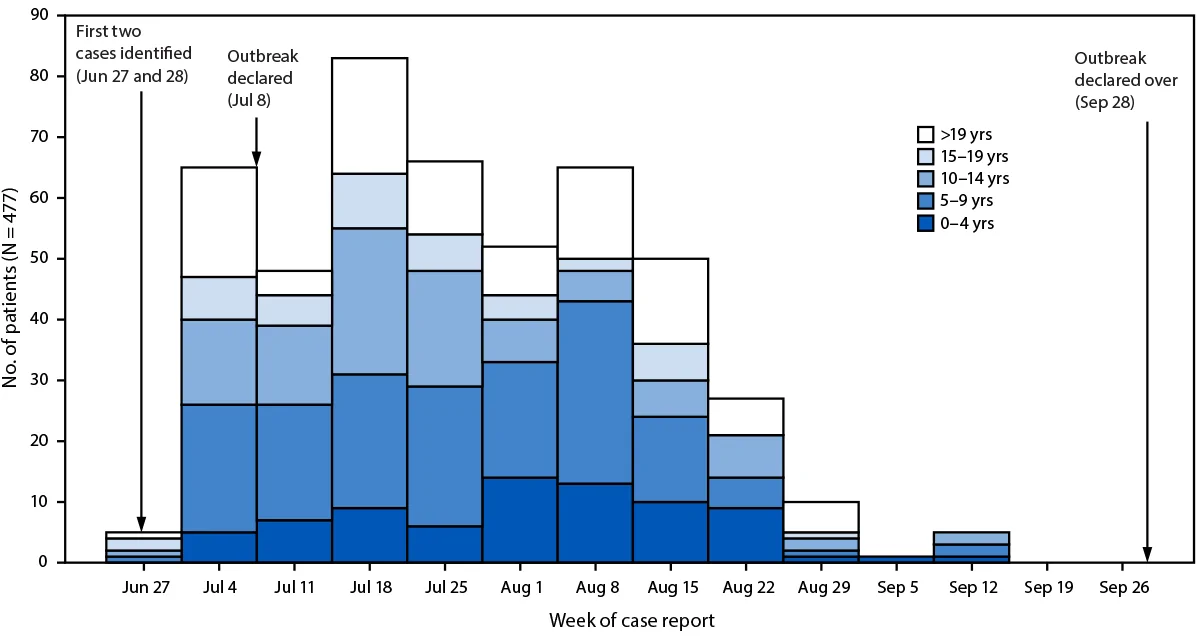
Number of patients with laboratory-confirmed dengue,* by age group and week of case report — Naoero, June 27–September 28, 2025 **Abbreviation**: IgM = immunoglobulin M. * Patients who received rapid diagnostic test results positive for nonstructural protein 1, IgM, or both were considered to have laboratory-confirmed dengue.

### Outbreak Response

**Case identification and clinical management.** To improve patient outcomes, health officials provided refresher trainings focused on strengthening timely case identification and delivering recommended clinical management. In response to hospital overcrowding, health officials established a dengue triage tent for rapid assessment of febrile patients and added a nine-bed dengue unit to the existing 65 inpatient beds. A majority of patients with suspected or confirmed dengue received care at home, with regular check-ups conducted through home visits; 114 patients were hospitalized during the outbreak. To address increasing demands for nursing staff members and medical supplies, the hospital suspended outpatient services and reassigned personnel and supplies from other areas of the hospital to dengue-dedicated areas.

**Public health messaging and vector control activities.** Public messaging on dengue prevention, including recommendations to use insect repellent and remove containers with standing water, and advice on when to seek care were broadcast through social media and other channels. To control mosquitoes in the community, vector control teams distributed larvicides and insect repellents; conducted indoor residual spraying in schools, households with persons with dengue, and neighboring residences; and held community campaigns to eliminate standing water and other places where mosquitoes breed.

## Preliminary Conclusions and Actions

This outbreak demonstrates the rapid and disruptive nature of dengue epidemics and the strain imposed on resource-limited health care systems. The disproportionate number of cases among young children, likely related to high levels of previous DENV exposure and immunity among adults, highlights the need to strengthen prevention and control strategies, including year-round vector control and clinical training for managing children with dengue. The outbreak further underscores the increasing need for safe and effective dengue vaccines, as well as for building and maintaining public health capacity to prepare and respond to the growing frequency and magnitude of dengue outbreaks and other public health emergencies in the Pacific Islands.

## Footnotes

* 45 C.F.R. part 46, 21 C.F.R. part 56; 42 U.S.C. Sect. 241(d); 5 U.S.C. Sect. 552a; 44 U.S.C. Sect. 3501 et seq.
